# Current Advancement of Immunomodulatory Drugs as Potential Pharmacotherapies for Autoimmunity Based Neurological Diseases

**DOI:** 10.3390/ph15091077

**Published:** 2022-08-29

**Authors:** Hajra Ashraf, Paolo Solla, Leonardo Atonio Sechi

**Affiliations:** 1 Department of Biomedical Sciences, University of Sassari, 07100 Sassari, Italy; 2 Department of Medicine, Surgery and Pharmacy, University of Sassari, 07100 Sassari, Italy; 3 Complex Structure of Microbology and Virology, AOU Sassari, 07100 Sassari, Italy

**Keywords:** the nervous system, autoimmunity, autoimmune-neurological diseases, immunotherapies

## Abstract

Dramatic advancement has been made in recent decades to understand the basis of autoimmunity-mediated neurological diseases. These diseases create a strong influence on the central nervous system (CNS) and the peripheral nervous system (PNS), leading to various clinical manifestations and numerous symptoms. Multiple sclerosis (MS) is the most prevalent autoimmune neurological disease while NMO spectrum disorder (NMOSD) is less common. Furthermore, evidence supports the presence of autoimmune mechanisms contributing to the pathogenesis of amyotrophic lateral sclerosis (ALS), which is a neurodegenerative disorder characterized by the progressive death of motor neurons. Additionally, autoimmunity is believed to be involved in the basis of Alzheimer’s and Parkinson’s diseases. In recent years, the prevalence of autoimmune-based neurological disorders has been elevated and current findings strongly suggest the role of pharmacotherapies in controlling the progression of autoimmune diseases. Therefore, this review focused on the current advancement of immunomodulatory drugs as novel approaches in the management of autoimmune neurological diseases and their future outlook.

## 1. Introduction

Immune system-related complications are not only confined to higher animals but faced by all types of life and overlooked by none. An unending and inexhaustible pressure is exerted by natural selection. The potential to shape the future of human history is dependent not only on previously emerging pandemics and epidemics but on continuously emerging infectious diseases. Control of these emerging diseases depends on the ability of our immune system to cope with these infections.

In autoimmune diseases, the functional and structural integrity of the definite cells, organs, and tissues becomes lost due to chronic immune reactions against the body’s cells, organs, and tissues. The prevalence of autoimmune neurodegenerative diseases is continuously increasing with time and, according to a study, the prevalence of multiple sclerosis (MS) increased up to 35.9 per ten thousand people in 2020 worldwide [1]. However, autoimmune diseases are not well understood, and a large group of diseases emerges from improper immune reactions against the body’s antigen. In most autoimmune diseases, inflammation was observed due to the association of autoantigen-specific T cells and antigen-presenting cells (APCs). Generally, inflammation and destruction of cells, tissues, or organs occur due to the absence of toleration against self-antigen and the emergence of autoantibodies in addition to the cellular immune response [2,3]. Autoimmune diseases can be classified into two groups (organ specific and systemic) based on their spread in the specific organ or into the entire body [4]. In organ-specific autoimmune diseases, the immune reaction is caused by autoreactive immune cells (B and T cells) that are limited to specific organs. Important organ-specific autoimmune diseases are multiple sclerosis (MS) [5], Crohn’s disease [6], vitiligo [7], type I diabetes [8], and psoriasis [9].

The central nervous system (CNS) has been regarded as an immune-privileged site due to the incapability of generating immune responses and the absence of innate immune response in the parenchyma of the CNS [10]. However, now studies suggest that immune cells are continuously accessing the central nervous system not only in disease patients but also in healthy subjects. A bunch of B cells can easily enter the parenchyma of CNS via crossing the perivascular space; however, in healthy subjects, the proportion of B cells in parenchyma is lower than in disease patients. Lymphocytes can also reach the parenchyma of CNS by crossing the thin fence of astrocytes called glia limitans. A study found the prevalence of T and B cells in healthy subjects’ CNS, confirming their role in immune surveillance [11].

Arlehamn et al. [12] confirmed the involvement of the immune system in the world’s most prevalent neurodegenerative diseases—Alzheimer’s and Parkinson’s diseases. A study found the association of the adaptive immune system in the progression of diseases. a-synuclein (a-syn)-derived T cells were found in the attacked region of Parkinson’s disease patients; moreover, T cells epitopes were also recognized in patients. Similarly, in Alzheimer’s disease, autoreactive T cells were also found to be localized in degenerative portions of the brain.

Neurodegenerative diseases are regarded as among the most challenging central nervous disorders, affecting almost 30 million people in the world. The pathophysiology of neurological diseases is complex but various studies confirmed the role of the immune system in the progression of neurological diseases. Therefore, the main therapeutic option to treat autoimmune neurological diseases is to suppress the autoimmune response either through cell-targeted immune suppression or by interference with immune cell activation and migration. In the current study, we aimed to provide a critical overview of recent data on pharmacotherapies used to treat autoimmune neurological diseases and to highlight the recent advances in drug development for neurological diseases. Moreover, this study underlines the potential drug candidates that are currently utilized to control autoimmune neurological diseases. This study may be used as a beneficial foundation to analyze the potential candidate drugs to improve the treatment outcomes of autoimmune neurological diseases.

## 2. Materials and Methods

This review is based on Google scholar and PubMed searches using the following keywords: autoimmune neurological diseases, immunomodulatory drugs, immunotherapies, multiple sclerosis, Alzheimer’s disease, neuromyelitis optica (NMO), Parkinson’s disease, and amyotrophic lateral sclerosis (ALS). Relevant articles were then investigated.

## 3. Results and Discussion

### 3.1. Immune System Mediated Neurological Diseases

#### 3.1.1. Multiple Sclerosis

Multiple sclerosis (MS) is the most common neurological autoimmune disease that is directly linked to T cells. It is most prevalent in young adults with multiple neuropsychiatric manifestations such as anxiety and depression. [13]. MS is referred to as a multiplex disease that relates to several infectious and environmental factors (i.e., Epstein–Barr virus (EBV), M. avium subspecies paratuberculosis (MAP), human endogenous retrovirus W(HERV-W), smoking, ultraviolet B light (UVB), obesity) along with multiple genes that moderately increase the vulnerability of the disease [14]. It is normally a two-phased disease with prompt inflammation that leads to relapse-remitting disease while later neurodegeneration results in non-relapsing disease development, i.e., secondary progressive MS (SPMS) and primary progressive MS (PPMS) [15].

MS pathophysiology is not completely understood, which makes it difficult to design an effective strategy to treat MS patients [16]. Currently, the major focus of MS treatments is to avert inflammations in the central nervous system (CNS). The first effective drug to treat MS patients was interferon-beta (IFN-beta), while many other drugs and monoclonal antibodies were used later such as natalizumab, fingolimod, glatiramer acetate, and alemtuzumab. Initially, all were found effective but several adverse effects were reported later, which makes their use difficult; various studies confirmed the unpromising effects of IFN-beta, i.e., stroke, migraine, headache, and depression. To date, various drugs are used for the treatment of MS and these are listed below [17,18].

#### 3.1.2. NMO Spectrum Disorder (NMOSD)

NMO spectrum disorder (NMOSD), also known as Devic disease, is an autoimmune neurological disease. NMO is associated with serum aquaporin-4 immunoglobulin G antibodies (AQP4-IgG). The clinical symptoms include lesions or syndromes of the optic nerve, spinal cord, area postrema, brainstem, and diacephalon [19]. The prevalence of NMO is lower in Caucasians but higher in Asian populations, approximately 48%. NMO mostly occurs at the age of 35–45 years but prevalence at an early age is also observed. The main pathogenic factor in NMO is the prevalence of AQP4-IgG in the blood that crosses the BBB and interacts with AQP4 accompanying activation of complementary molecules and induction of antibody-dependent toxicity, which results in damage of astrocytes. Activation of complementary molecules along with cytokines activates other inflammatory molecules which enhance the BBB damage and increase the entry of AQP4-IgG in the brain [20,21].

#### 3.1.3. Alzheimer’s Disease

Among the leading causes of dementia in elderly people is Alzheimer’s disease (AD). It is known for its obvious symptoms including a continuous decrease in cognitive abilities, changes in psychotic and behavioral characteristics, and alternatively a drop in patient autonomy and death [22]. Through the pathology of the nervous system, it is obvious that the main reasons behind this are intraneuronal neurofibrillary tangles (NFTs) and aggregated amyloid β-peptide plaques [23,24]. However, despite NFTs and amyloid β plaques, extended evidence suggests the role of autoimmunity in AD via mechanistic studies, genome-wide association, and clinical correlation [25]. Autoimmunity in AD may arise due to pathogen mimicry of AD-related pathogens or via downregulation of self-tolerance mechanisms [26,27].

Mutations also play an important role in AD pathogenesis. Less than 5% of mutations cause the severity of this disease, but they have a great impact on the development of AD. The more common mutations are observed in the presenilin 1 (chromosomal 14), presenilin 2 (chromosome 1), and amyloid precursor protein (APP) genes (chromosome 21). Epidemiological studies also confirmed that educational level, head injury, and other factors also contribute to the development of AD [28,29].

#### 3.1.4. Parkinson’s Disease

Parkinson’s disease (PD) is regarded as the second most prevalent neurodegenerative disease in the world after AD [30]. Presenting both with motor and non-motor symptoms, the disease is more common in older age, but some cases are also seen at a younger age [31]. The major clinical symptoms of Parkinson’s disease are bradykinesia, resting tremors, rigidity, and, in advanced stages, postural instability [32]. Among the significant hallmarks of this disease is dopamine deficiency, which results in the death of dopaminergic neurons [33]. Several non-motor symptoms are also observed in PD patients, including sleep disorders, cognitive impairment, loss of olfaction, constipation, and depression even before onset of motor symptoms [33,34]. In most cases, PD remains without any obvious cause but mutations in certain genes are associated with the onset and severity of diseases, i.e., PTEN (phosphatase and tensin homolog)-induced putative kinase 1 (PINK1), α-synuclein(α-syn), leucine-rich repeat kinase 2 (LRRK2) and parkin [35]. Although the pathophysiology of the diseases is not clearly understood, the interaction between certain gene products such as α-syn, PINK1, parkin and immune cells (i.e., B cells, T cells, dendritic cells, microglia, etc.) has been found to be involved in the pathogenicity of disease [36].

#### 3.1.5. Amyotrophic Lateral Sclerosis (ALS)

Amyotrophic lateral sclerosis (ALS) also known as Lou Gehrig’s Disease is a fatal, progressive, and most frequent adult-onset neural disease characterized by the progressive death of motor neurons, leading to global paralysis and ultimately death of the patient. The prevalence rate of this disease is 6–8 × 10^5^, while the incident rate is 2 × 10^5^. Studies confirmed that various non-neuronal cells contribute to the severity and progression of the disease such as astrocytes, microglia, T, and muscle cells [37].

## 4. Factors Affecting Neurodegeneration

Multiple factors are responsible for neurodegeneration including environmental, age, and genetic factors.

### 4.1. Age

Aging is a complicated natural biological process that imparts a major role in most prevalent disorders, i.e., cancer, heart diseases, and neurological diseases such as Parkinson’s and Alzheimer’s diseases. Deterioration of neuroendocrine and immune systems leads to neurological diseases by creating oxidative stress, dysregulation of neuronal transmission, alteration in brain proteostasis, and impedes the functionality of mitochondria and energy metabolism. At a particular age, there are chances of tau and a-synuclein hyperphosphorylation, amyloid plaques formations, and an increase in the accumulation of transactive DNA binding protein 43 (TDP-43) that ultimately leads to alteration in brain morphology and loss in the brain and neuronal volume [38]. These variations in the brain contribute to the development of multiple autoimmune-neurological diseases (AINDs) [39].

### 4.2. Environment

The etiology of most neurological diseases is still unknown; therefore, in numerous cases, environmental factors represent a key element in the development of these diseases. Factors involved include pesticides, microorganisms, drug abuse, metals, viral and bacterial infections [40].

### 4.3. Genetic Factors

Changes on the genetic level have little effect on the progression of the disease. Certain changes in gene outcomes enhance susceptibility to inheritable diseases. Various studies confirmed the association of genetic changes with numerous neurodegenerative diseases by investigating the molecular mechanisms of a particular disease. A powerful tool used for this kind of study is genome-wide association studies that confirm the relationship of genetic changes with various neurodegenerative diseases [41].

#### Immunomodulatory Drugs

Immunomodulatory drug development is increasing day by day. These drugs modify the immune system either by decreasing (immunosuppressives) or increasing (immunostimulators) serum antibodies [42]. In 1998, the US Food and Drug Administration first approved thalidomide as an immunomodulator for the treatment of leprosy. Pomalidomide was approved in 2013 for the treatment of refectory or relapsed myeloma while Lenalidomide was approved for the treatment of multiple myeloma in 2015 [43]. To date, many immunomodulatory drugs have been approved; however, certain advantages and limitations are associated with them (Table 1).

## 5. Potential Pharmacotherapies for Autoimmune Neurological Diseases

### 5.1. miRNAs as a Potential Biomarker for Neurological Diseases

Modern pharmacology is greatly impacted by major and rapid advances in the field of medicine, thus generating a new field known as pharmacogenomics [51,52]. Herein, the field genome-wide study of each individual is performed to analyze the variations in drug response due to epigenetic factors. Three interrelated molecular mechanisms come under genome-wide studies, i.e., DNA methylation, histone modification, and gene regulation via non-coding RNAs such as circular RNAs, long non-coding RNAs, and miRNAs [52].

**Figure 1:**
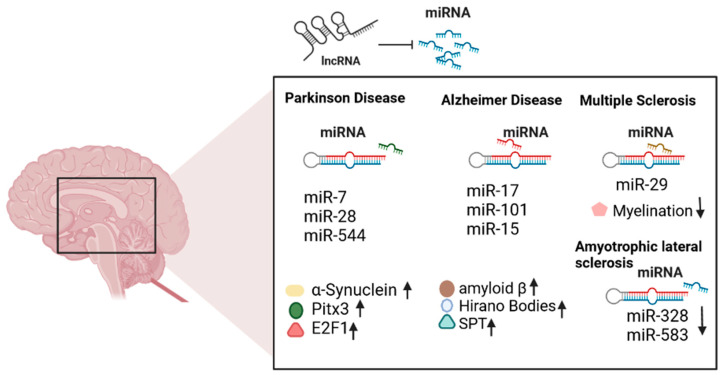
MicroRNA dysregulation in neurodegenerative diseases. 1. Dysregulation of microRNAs results in an increased level of α-syn and other transcriptional factors such as Pitx3 and E2F1in PD. 2. Numerous miRNAs contribute to the pathogenic pathways of AD, i.e., miR-17 is involved in APP splicing, miR-101 is involved in neuroinflammation, and miR-15 is involved in apoptosis. Disruption in miRNA results in aggregation of amyloid-β, Hirano bodies, and serine palmitoyltransferase (*SPT*) levels. Various miRNAs are also involved in α-syn aggregation, i.e., miR-7, while others are involved in oxidative stress such as miR-28, and some are associated with mitochondrial functions such as miR-544. 3. Dysregulation of miRNAs is also reported in MD, which results in increased demyelination. 4. Downregulation of miR-328 and miR-583 slows down the progression of ALS.

Recently, post-transcriptional regulation by microRNAs (miRNAs) has gained more attention as a newly developed mechanism. In many AINDs, dysregulation in miRNAs is observed (Figure 1). Therefore, therapeutic effects of miRNAs have been proposed by two models, i.e., direct and indirect regulations. In the direct model, the altered miRNA expression level is restored directly by neurodegenerative diseases approved drugs [53], while the indirect method suggested that miRNA may affect the gene expression that contributes to drug absorption, distribution, metabolism, and excretion (ADME), thus enhancing the drug efficacy. Through epigenetic regulation of miRNAs in ADME genes, it can be justified why variation in drug response is seen among different individuals. Considering the multiple factors that are responsible for creating inter-individual differences in the efficiency of given drugs is necessary to develop personalized medicine [54,55].

Currently, the majority of treatments for MS patients are administered to lower the relapses and decrease the progression of disabilities [56,57]. In this context, there are multiple disease-modifying therapies (DMTs) that are used to repress or regulate the immune system, overcome relapses, prevent CNS lesions, and restrain inflammation in CNS [56].

Research conducted by various studies confirmed that, through disease-modifying therapies, dysregulation in miRNAs can be restored. More than 20 years ago, glatiramer acetate (GA) and interferon-β (IFN-β) were frequently used as DMTs for MS patient treatment. IFN-β proved an effective therapy because of its capability to regulate the immune system by decreasing the movement of T lymphocytes towards the central nervous system [58]. The first study to determine the effect of IFN-β on miRNAs regulations found that overexpression of IFN-β-related genes was directly related to the suppression of miRNAs, i.e., miR-29 family in peripheral blood mononuclear cells (PBMCs) of MS patients [59]. In another study, it was proved that administration of IFN-β therapy results in the normalization of miR-145 and miR-20a-5p overexpression [60]. Similarly, GA administration was also found effective in normalizing the miR-142-3p and miR-146a levels in Primary Peripheral Blood Mononuclear Cells (PBMC) of MS patients [61]. Singh et al. [62] illustrated in their study that GA administration in MS patients results in a balance of miR-27a-3p, miR-350-5p, miR-155-5p, and miR-9-5p levels in urine exosome and plasma confirming it an efficient drug biomarker.

In another study conducted by Muñoz-Culla et al., it was found that the expression level of three miRNAs (miR-629, miR-320b, and miR-320) was modified in MS patients after the treatment with Natalizumab [63]. The linkage of genetic variants of miR-146a with MS patients was confirmed by Yuan Zhou et al. In this study, the functional polymorphism in the miR-146a gene was investigated to identify the linkage with MS patients. The results recognized the linkage of EBV infection and miR-146a regulation with MS [64].

Like MS, the complete etiology of PD is still not known; however, both environmental and genetic factors contribute to the progression of the disease. To date, there is no effective biomarker for this disease; however, certain studies suggested the association of miRNAs with Parkinson’s disease pathophysiology as miRNAs were found to be involved in the regulation of various PD-related genes [35,65].

Although an effective treatment to stop neurodegeneration in AD has not yet been proved, some drugs are used to improve and stabilize AD symptoms and enhance the quality of life of patients. There are two kinds of drugs that are usually used for AD: one is cholinesterase inhibitors (i.e., rivastigmine, donepezil, and galantamine), while the other is memantine, an N-methyl-D-aspartate (NMDA) receptor antagonist [66]. Wang et al. [67] studied the effect of donepezil on the expression level of miR-206-3p. With the administration of donepezil, a decrease in the expression level of miR-206-3p was observed, suggesting that miR-206 could be a possible target for developing novel therapies for AD as a higher level of miR-206 was associated with downregulation of brain-derived neurotrophic factor (BDNF). Apart from conventional drugs, some plants’ secondary metabolites also have a positive effect on miRNA expression levels that can influence the pathogenesis of AD [67]. Osthole is a derivative of coumarin naturally extracted from *Cnidium monnieri* (L.) and various studies proved the pharmacological role of osthole (i.e., osteogenesis, anti-apoptosis, neuroprotection, anti-oxidation, and anti-inflammation) [68,69]. Although the neuroprotective effect of osthole is observed in AD patients, it is not clear how it influences AD but several studies suggested the possible involvement of miRNAs.

Li et al. [70] found that on the application of osthole, the expression of miR-9 was significantly high imparting a positive role on a neuronal synapse. In another study conducted by Li et al., the authors observed the association of miR-9 with the notch signaling pathway as administration of osthole upregulates the miR-9 which promotes Neural stem cells (NSCs) differentiation and inhibits the notch signaling pathway. A similar study was conducted by Jiao et al. in which they found an association of osthole with overexpression of miR-107 in AD [35,68].

Apart from the role of miRNAs in MS, PD and AD studies also found the association of miRNAs in ALS. Riluzole is the only FDA-approved drug currently used for ALS to slow down the progression of the disease and enhance the survival rate. Currently, the major focus is to investigate the potential role of miRNAs in the etiology of ALS disease. A study conducted by De Felice et al. for the first time confirmed the deregulation of eight miRNAs (miR-149, miR-451, miR-338-3p, miR-1275, miR-665, miR-328, miR-583, and miR-638) in ALS patients by extracting the leukocytes from both healthy and affected individuals. Shioya et al., 2010 [71] previously also confirmed the presence of miR-338-3p in the brain of patients. Another study conducted by Butovsky et al. [72] found a higher level of miR-32-3p, miR-27a, miR-146a, and miR-155 in ALS patients.

Thus, the data obtained identify the miRNAs as potential biomarkers for AINDs but still, there is much more to explore as, although the involvement of miRNAs is confirmed, no possible solution is yet proposed considering miRNAs [73]. Moreover, if we can improve our understanding of the pathogenesis of AINDs, this could lead to the development of early and specific diagnostic methods and extend the life expectancy of AINDS patients.

### 5.2. Phytochemicals for the Treatment of Autoimmune Neurological Disease

**Figure 2:**
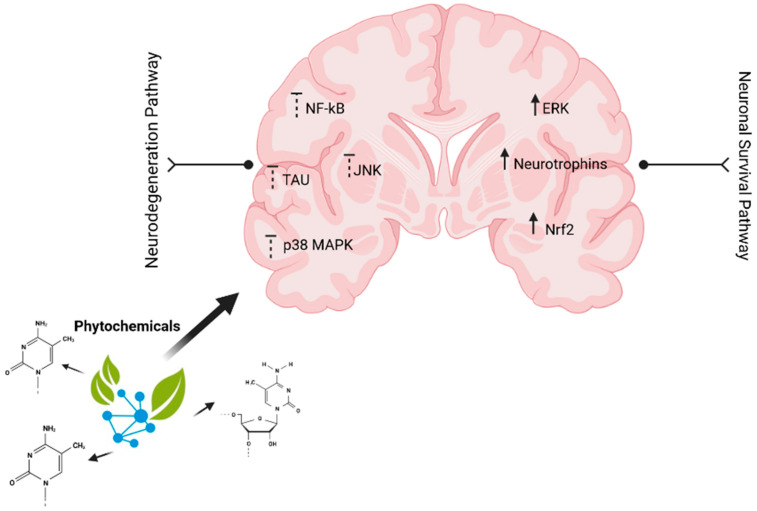
Phytochemicals: mechanism of action against Immune neurodegenerative diseases.

Among the major causes of neurological disease is inflammation, which leads to neuronal damage. Certain pro-inflammatory mediators are released by microglia, resulting in neuroinflammation, which ultimately results in neurodegeneration. The proinflammatory mediators include tumor necrosis factor-alpha, leukotrienes, free radicals, and cytokines. The proinflammatory molecules activate different signal transduction pathways including mitogen-activated protein kinase (MAPK), phosphoinositide 3-kinase/protein kinase B (PI3K/AKT), and mammalian target of rapamycin. These pathways ultimately activate various transcriptional factors including hypoxia-inducible factor-1 alpha (HIF-1a), nuclear factor kappa-light-chain-enhancer of activated B cells (NF-kB), and various signal transducers, which ultimately results in inflammatory responses. Phytochemicals act as neuroprotective agents by targeting or inhibiting the inflammatory mediated pathways (Figure 2).

#### 5.2.1. Flavonoids

Flavonoids are naturally occurring phytochemicals found abundantly in vegetables, fruits, and numerous beverages. These are secondary metabolites that contain polyphenolic rings and play a major role in plant color and help in the plant defense system by acting against microbes. Various studies confirmed the antioxidant, antimicrobial and anti-inflammatory potential of flavonoids that make them a potential candidate for therapeutic purposes [74]. The antioxidant potential of flavonoids helps to scavenge the free radicals and upregulate the antioxidant defense system; in addition, flavonoids help to compete with the enzyme that is involved in inflammatory processes to overcome inflammation [75]. These attributes of flavonoids contribute to decreasing neural damage and hamper neurodegenerative disease progression [76].

##### Quercetin

Quercetin is a naturally occurring flavonoid commonly found in mulberry fruits. It has strong antioxidant potential and, therefore, helps to scavenge oxidative free radicals and increase neuroprotection. Due to this activity, quercetin was found to upregulate the neuronal survival rate [77,78].

##### Apigenin Derivatives

Apigenin derivatives are flavonoids abundantly found in Passiflora plant species. Numerous studies confirmed the neural protective role of apigenin. Apigenin derivatives exert antioxidant and anti-inflammatory effects by direct influencing lipopolysaccharide-activated microglia and inhibiting nitric oxide and prostaglandin 4 production, thus influencing the neuroprotective role [79]. Furthermore, apigenin has permeability toward the blood–brain barrier and, therefore, serves as an effective secondary metabolite for neurodegenerative diseases related treatments [80].

##### Diosgenin

Diosgenin is a sapogenin steroid that is a major component of *Dioscorea nipponica* and is widely used as a medicinal plant for the treatment of neurodegenerative, diabetes, and various inflammatory diseases. Sapogenin is found to induce strong nerve growth factor (NGF) expression, expanding the outgrowth of neuronal cells and reduction in free radical production, i.e., nitric oxide production [81]. Studies confirmed the role of diosgenin in the upregulation of NGF secretions, provoking neural regeneration and enhanced nerve conduction velocity [82].

##### Rosmarinic Acid

Rosmarinic acid is a natural polyphenol commonly found in *Melissa officinalis* and exerts a neuroprotective effect by stimulating cholinergic activity and enhancing cell differentiation. The neural protective role of rosmarinic acid is due to its activity to suppress overexpression of hypoxia-inducible factor-1α (HIF-1α), which is involved in the induction of hypoxia-induced proinflammatory cytokines and caspase 3 activity; moreover, it enhances memory by upgrading cholinergic activity [83].

## 6. Gene Therapy as a Potential Treatment Strategy for Autoimmune Neurological Diseases

In the current world, gene therapy has gained much attention due to the effective transfer of genetic material into the targeted cell to rectify the defective gene. Among the major advantages of this technique is that it ensures a long-lasting restorative effect [84]. Gene therapy rectifies the defective gene either by inserting a new copy of the gene or utilizing gene-editing technology. There are two vectors for gene therapy—viral or non-viral. The immunogenicity concern is lower with nonviral vectors in comparison to viral vectors [85]. Previously, gene therapy was normally used for monogenetic disease treatment; however, considering the different environmental and genetic factors, gene therapy is also utilized for autoimmune neurodegenerative diseases [86]. Autoimmune diseases are due to pro-inflammatory reactions generated due to the body’s own autoreactive cells. Various researchers aimed to use gene therapy to investigate the mechanism to silence autoreactive cells to control autoimmune disorders.

Gene therapy that involves chemokines and cytokines is currently investigated in EAE mice. Sloane et al. [87] investigated the effect of IL-10 gene therapy in the experimental autoimmune encephalomyelitis (EAE) rat model by injecting the plasmid under the control of a hybrid promoter named cytomegalovirus enhancer/chicken beta-actin that encodes IL-10 complementary DNA (cDNA) of the rat. A study conducted by Geoffrey D. et al. [88] developed a robust and safe immune-modulatory therapy using adeno-associated virus (AAV). In the study targeting the liver, a gene-transfer vector was designed that can express myelin oligodendrocyte glycoprotein (MOG). Results found that by inducing MOG-specific regulatory T cells (Tregs), immune tolerance in mice was restored and mice remained protected from neurological deficits and developing disease.

In multiple sclerosis, myelin basic protein (MBP) has been regarded as a potent autoantigen. Evidence via various research suggested that antibodies associated with MBP, interferon-gamma, cytokines, and T cells are mainly involved in the development of disease. Tolerance or downregulation of antigenic response to MBP could be a possible solution for MS patient treatment [89]. In the EAE mouse model of MS, it was observed that injection of a plasmid containing the encephalitogenic T cell epitope repressed interferon-gamma and reduced the histopathological and clinical symptoms of EAE [90,91]. Similarly, Yoo et al. [92] studied the effect of the anti-inflammatory cellular immune response elements (CIRE) gene in an AD mouse model of amyloid-β protein precursor (AβPP). The result found neuroinflammation downregulation and decline or delay in cognitive response in AD mouse model on the application of CIRE gene therapy.

## 7. Nanotechnology for Autoimmune Neurological Diseases

Nanoparticles are particles that range between 1 and 100 nm and are not less than one dimension. In the current world, nanoparticles have gained attention due to their effective use in the field of medicine, specifically in therapeutics and diagnosis [93]. A broad range of materials can be used to synthesize nanoparticles including metals, polymers, or carbon sources. The high effectiveness of nanoparticles is associated with their size, surface-to-volume ratio, and shape. Moreover, these properties can be enhanced by attaching various capping and stabilizing molecules to the surface of nanoparticles [94].

Currently, nanomaterials including quantum dots, nanotubes, nanoparticles, and nanofibrils have broad-spectrum applications in the field of medicine such as in bioimaging, drug delivery, and biosensors. Neuro-nanomedicine is a newly emerging field in nanotechnology in which nanoparticles are engineered to treat neurodegenerative diseases [95,96]. Nanotechnology aims to engineer nanoparticles in such a way that they act as nanoscale devices to interact with biological entities at a molecular state. The engineered nanodevices aim to interact, trigger and generate a response from the target cells or tissues having the least side effects. The major hurdle in the treatment of neurodegenerative disease is that some drugs cannot penetrate the blood–brain barrier, which affects the efficacy of drugs; therefore, some nanoparticles are designed in such a way that they can easily cross the blood–brain barrier to reach the specific target site in the cells [97,98]. Presently, the major focus is on the development of nanoparticles that are easily localized intracellularly or outreach to target extracellular molecules such as amyloid-beta plaques in AD [99].

Some of the nanoparticles used in the treatment of autoimmune neurodegenerative diseases are mentioned below

### 7.1. Gold Nanoparticles

Gold nanoparticles are regarded as theranostic nanoparticles due to their major applications in the field of surface modification, therapeutics, and imaging [100]. M. Sanati et al. [101] confirmed that gold nanoparticles in amalgam with exosome-derived membranes have efficient target-specific delivery to the brain. Furthermore, bioluminescence imaging studies confirmed that exosomes coated with gold nanoparticles reach the brain-targeted cells efficiently. The modification of the surface through gold nanoparticles makes targeted exosome delivery and can be proved as effective and novel strategy for targeted delivery.

Amyloid disorders are associated with amyloid plaques formations along with the production of various toxic materials that result in amyloid diseases such as Alzheimer’s disease. The disease is due to not proper folding of some functional proteins and peptides. Thus, to treat amyloid disorders, the major focus was on the development of therapeutics that dissociate, inhibit, or delay the amyloid fibrils formation. To determine the effect of gold nanoparticles on amyloid formation, α-lactalbumin was used as a potential candidate for amyloid formation the study confirmed the potential effect of AUNPs on inhibition of amyloid fibrils due to higher absorption of NPs on protein and prevention of structural changes [99,102]. Thus, it can be an effective agent for the treatment of amyloid diseases.

### 7.2. Magnetic Nanomaterials

Magnetic nanoparticles (MNPs) are among the most focused nanoparticles in various fields including biotechnology, magnetic resonance [103], biomedicine [104], catalysis, environmental remediation [105], magnetic fluids [106], and data storage [107]. Currently, MNPs have gained much importance in the field of medicine owing to their magnetic force response potential that has an influential impact on cell sorting and targeted drug delivery. Magnetic targeting has the concept to load the potential drugs within the magnetic nanoparticles so that magnetic field gradients localized the drug at target site effectively to minimize higher dose side effect and lessen the nanoparticles toxicity. In the development of nanomaterial-based therapies, the size of nanoparticles seems important parameter. To date, small-sized nanoparticles were found to be effective in targeted drug delivery, easy control over the direction of nanoparticles and higher drug localization at target site. Currently, a supramagnetic nanoparticle with a core of iron nanoparticle is of great interest as it can easily be made compatible with various functionalized drugs for specific targeting drugs [108,109].

### 7.3. Cerium Oxide Nanoparticles

Cerium nanoparticles are also known as nanoceria. They are regarded as multifunctional nanopolymers that have high catalase or dismutase activity and higher bioavailability. The antioxidant potential of nanoceria is also high due to their activity to scavenge free radicals effectively [110]. The study showed that nanoceria has neural protection property against mitochondrial fragmentation induced by amyloid beta protein and decrease Ser616 hyperphosphorylation which is strongly associated with neurodegenerative diseases, particularly Alzheimer’s disease [111]. Another study depicted the positive influence of nanoceria on the Parkinson’s disease yeast model. Yeast cell viability significantly increases after the implementation of cerium oxide nanoparticles due to *Alpha*-synuclein (α-syn) expression. The enhanced viability of yeast cells is directly associated with nanoceria interaction with α-syn as these nanoparticles prevent α-syn aggregation and reduced ROS production [112].

### 7.4. Graphene Quantum Dots (GQDs)

Graphene quantum dots (GQDs) are made up of a single or few layers of graphene having a size of less than 100 nm. Owing to their higher biocompatibility and lower toxicity GQSDs have immense potential in the field of biomedicine [113]. Kim et al. [114] illustrated that GQSDs easily crossed the blood–brain barrier and lessen α-syn fibrils formation through direct interconnection with mature fibrils. Despite of that GQSDs did not cause any toxicity both in vitro and in vivo, decreased neuronal death, prevent neuronal transmission of dysregulated α-syn, and alleviate dysfunctional and damaged mitochondria. GQDs are also used in Alzheimer’s disease to control the aggregation of Aβ. the β-amyloid peptide is made up of several amino acids comprising several regions in which the His13-Lys16 (HHQK) region plays a crucial role in fibril formation and oligomerization. It is also regarded as an important component of the binding site of glycosaminoglycan (GAG). Construct consisted of GAGSs mimic named as tramiprosate and GQDs showed a decline in Aβ aggregation by β-sheet breakdown. Similarly in another study, the same construct exerts a synergistic effect and protects the PC12 cells from cytotoxicity of Aβ [115,116].

## 8. Stem Cells Based Neurotherapies

Currently, autoimmune neurodegenerative disease treatment through stem cells is the most promising field as neuronal cell degeneration and regeneration in an adult human being is in equilibrium [117]. Although neurogenesis in an adult is still a question, it can be improved by stem cells as they have the potential to differentiate in any cell. Despite different neurodegenerative diseases having different pathophysiology, cognitive impairment is common in all that is directly related to synaptic function loss. Therefore, cognitive impairment can be reduced with replacement and regenerative therapy through stem cells [118]. The most promising stem cells used for neurodegenerative diseases are embryonic stem cells, neural stem cells, mesenchymal stem cells, and induced pluripotent stem cells [119].

### 8.1. Neural Stem Cells (NSCs)

Neural stem cells (NSCs) can be obtained from the patient’s embryonic or somatic stem cells. They can differentiate into neurons, astrocytes, and oligodendrocytes [120]. NSCs differentiation needs careful control through signaling pathways to clarify the NSCs fate both in situ and in vivo. Currently, in a phase one clinical trial, NSCs have been implanted into the spinal cord of ALS patients exogenously to slow down the disease progression [121]. Moreover, studies show the beneficial impact of human neural stem cells in a mouse model of Alzheimer’s disease. In AD mouse models, the human neural stem cell line improved cognitive behavior by boosting synaptogenesis endogenously. Synaptic markers, i.e., synapsin, growth-associated protein-43 (GAP-43) and synaptophysin also increases in AD models due to successful differentiation of transplanted cells into immature glia and neuronal cells. Despite of that reduction in pathology of tau and Aß was not observed, suggesting the role of NSCs in just maintaining the degeneration and not treating the pathology of disease [118]. Research conducted by McIntyre, L. L et al. confirmed that the plantation of neural progenitor and neural stem cells(NPCs and NSCs) in EAE mice results in increased myelination and dampened neuroinflammation due to the emergence of regulatory T cells (Tregs) [122].

### 8.2. Embryonic Stem Cells (ESCs)

Embryonic stem cells (ESCs) have great importance in the medical field due to their self-renewal and totipotency nature. Nevertheless, various medical, religious, and ethical concerns limit the usage of embryonic stem cells in NDD. There are certain limitations associated with the ESCs such as immune system rejection by host cells due to allogenic sources [123] and enhancing the risk of tumor formation or cancer development due to rapidly migrating and dividing ability [124,125]. However, current findings on mouse models show the higher potential of ESCs in the formation of dopaminergic neurons which is not achieved through NSCs. In Parkinson’s disease, dopaminergic neurons are very crucial for treatment as they can easily coordinate with the neuronal system [126]. Moreover, the study confirmed that ESCs exhibit strong potential to migrate into the spinal cord and parenchyma and can partially recover motor neurons in mouse models having spinal cord injury. Hence, ESCs can be a potential source for fully recovering degeneration of motor neurons in PD [127].

### 8.3. Induced Pluripotent Stem Cells (iPSCs)

In 2006, induced pluripotent stem cells (iPSCs) were discovered by reprogramming somatic cells into embryonic-like pluripotent stem cells and from that day incredible progress has been made [128]. Through iPSCs, the problem of auto rejection by patient immune cells is eliminated as patients’ reprogrammed stem cells can be used for plantations; moreover, there is no ethical or religious concern over the use of iPSCs [129]. Human iPSCs can be easily transformed into dopaminergic neurons; however, direct transformation into DN is not possible and, therefore, before differentiation, it must be cultured to the appropriate progenitor stage. If iPSCs are implanted in the undifferentiated state, they mostly form tumors; therefore, this is a major concern in their application in neurodegenerative diseases and reprogramming through viral vectors may lead to viral incorporation in iPSCs, causing various mutations and chromosomal disruptions. Currently, non-viral vectors are being implemented to improve iPSC reprogramming safely and effectively [119,130].

### 8.4. Mesenchymal Stem Cells (MSCs)

**Figure 3:**
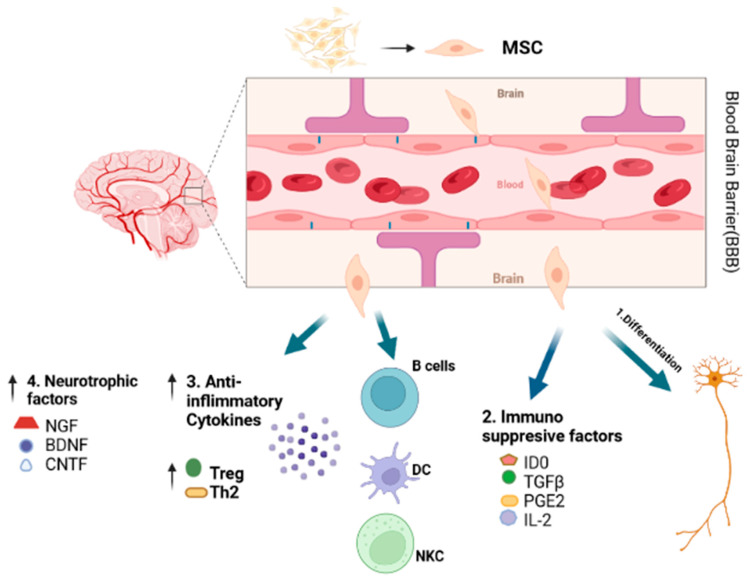
Mechanism of action of the mesenchymal stromal cell (MSC) against autoimmune neurological diseases. 1. MSCs regenerate into neuronal cells. 2. Release of neuroinflammation factors. 3. Activation of immunosuppressive factors such as ID0, TGFβ, PGE2, and IL-2. 4. Increase in the release of anti-inflammatory cytokines, i.e., Treg and Th2. 5. Secretion of neurotrophic factors, i.e., BDNF, CNTF, and NGF, helps in the differentiation of neuronal and glial cells. IDO = indoleamine 2,3-dioxygenase, TGFβ1 = transforming growth factor β1, PGE = prostaglandin, IL2 = interleukin 2, Treg = regulatory T cells, TH2 = T helper 2 cells, BDNF = brain cell-derived neurotrophic factor, CNTF = ciliary neurotrophic factor, and NGF = nerve growth factor.

Mesenchymal stem cells (MSCs) are multipotent, adult, self-renewal cells that can develop fat, cartilage, bone, and epithelial cells in vivo and be differentiated into glial and neuronal cells in vitro after differentiation (Figure 3). MSCs can be collected from adipose tissues, umbilical cord, spleen, and bone marrow that make it easy to harvest from patients. After harvesting, MSCs can be differentiated into glial and neuronal cells and easily implanted into the central nervous system. The main function of MSCs is a synthesis of neurotrophic factors to activate neuroregeneration by activating microglia and stimulating neurogenesis, which results in more Aß plaques clearance. MSCs also enhance angiogenesis and neural progenitor cell recruitment through the secretion of angiogenic cytokines, stromal-derived factor 1 (SDF1), and angiopoietin-1. Placenta-derived mesenchymal stromal cells (PDMSCs) were found effective in the treatment of MS when delivered either at the onset or peak of the disease. PDMSCs exerted neurotropic support in MS patients through neurotrophin expression [131].

## 9. Peptides as Potential Neurotherapeutic Agents 

With extensive research regulated by various pharmaceuticals and biotechnological companies, a vast class of therapeutics has emerged. Oxytocin was the first synthetic peptide discovered in 1953; later, recombinant DNA technology permitted the vast production of peptides on an industrial scale. The current advancement in the field of synthetic peptides has developed a greater interest in industries and scientific communities to identify and develop therapeutic peptides. Owing to low cost, higher specificity, more membrane perforation ability, and much specificity, peptides have more advantages over various molecules at the molecular level. Nonetheless, while developing peptides immunogenicity, stability and toxicity remain a major concern [132,133]. According to a study, it is estimated that an extensive percentage of world disabilities and deaths are associated with neurological diseases therefore peptides proved a beneficial tool to study the effects associated with misfolded proteins or peptides.

### 9.1. Carnosine

Carnosine is a natural polymer composed of alanine and histidine and has the strong antioxidant potential [134]. It is normally found in human nervous tissues and the brain easily crosses the blood–brain barrier and is frequently absorbed in the human digestive system. Carnosine is regarded as a hydrophilic molecular of lower weight that actively participates in free radical scavenging activity [135]. It is a major constituent of dietary foods such as rabbit, beef, tuna, chicken, and turkey. Various studies suggested that taking carnosine as a dietary supplement helps to cure various NDDs, i.e., Parkinson’s disease [136], multiple sclerosis [137], and Alzheimer’s disease [138,139].

### 9.2. P110

Various studies suggested the association of dopaminergic neuronal cell death with mitochondria-mediated pathways in PD. Dynamin-related protein 1 (DRP1) found greater command over mitochondrial fission. Under stress conditions, it is found that DRP1 becomes activated and moved towards mitochondria, which results in mitochondrial fission which ultimately results in dopaminergic neuronal cell death. Emily Filichia et al. suggested that P110 a peptide inhibitor results inhibition of DRP1 which helps in dopaminergic neuron protection of PD patients [140].

### 9.3. Vasoactive Intestinal Peptides

Vasoactive intestinal peptides are neuropeptides consisting of 28 amino acids. VIPs are generally associated with the glucagon family that has wide applications in various biological systems [141]. Numerous researchers suggested the protective role of VIPs in different autoimmune neurodegenerative diseases. Through various in vivo and in vitro studies, it is found that during the pathogenesis of various autoimmune neurodegenerative diseases (i.e., MS, PD, and AD) VIPs play a crucial neuroprotective role [142,143,144].

## 10. Etiological Concerns Related to Autoimmune Neurological Diseases (AINDs) Therapies

### 10.1. Novel Etiological Molecular Biomarkers in AINDs

There are etiological concerns related to AINDs, and studies found the association of microbial infections with numerous autoimmune neurological diseases [145]. Various bacterial and viral species can colonize in tissues and organs of the body including the brain [146]. Early detection of microbial infections might be difficult due to their slow progress, entailed antibodies, fewer burden, and various sensitive DNA-based assays. The determination of the central nervous system (CNS)-affecting region can be possible through specific infecting microorganisms, their way of entrance, and the response of each microbial species. Microbial infections can trigger an autoimmune response by including multifarious immune system pathways. Moreover, with the constant release of extracellular enzymes (lipases, proteases, etc.) and toxic metabolites, brain atrophy and destruction of neuronal cells were observed. Eventually, the microbial infection can spread progressively to other neuronal cells, leading to clinical symptoms evaluation. Significant in vivo and in vitro studies confirmed the immune response linkage with microbial infections and the evolution of autoimmune diseases, i.e., MS, and ALS [147,148]. There is also evidence for the role of gut microbiota dysbiosis in the severity and pathogenesis of autoimmune neurological diseases.

Recent studies indicated human endogenous retrovirus K (HERV-K) as a possible etiological agent for AINDs. To date, human endogenous retroviruses (HERVs) are considered junk DNA, constituting approximately 8% of total genomic DNA. Integration of the pro-viral genome into human DNA is an indication of viral infection taken place many years ago. However, various mutations are considered to be responsible for their abscond [149].

HERV-K is regarded as the most transcriptionally active HERVs that are present in more than a hundred copies of the human genome. Various groups documented the involvement of HERV-K in ALS pathophysiology. The study found the altered expression level of HERV-K transcripts in the brain of ALS patients in comparison to other neurological diseases, i.e., PD. Similarly, motor dysfunction was observed in transgenic mice in which the envelope (env) gene was inserted [150].

In a recent study, humoral response was investigated against four HERV-K antigenic peptides in cerebrospinal fluid (CSF) and serum of ALS patients. A notable immune response was observed against HERV-K peptides due to transcriptional activation and the toxic effect of HERV envelope (env) protein. Additionally, an active IgG antibody production was found within the central nervous system [151]. Arru, G. et al. [152] found the higher production of HERV-K on expression onto natural killer (NK) and B cells of ALS patients. In the study, the association of HERV-K with ALS was confirmed by cytometric analysis, reverse transcriptase-polymerase chain reaction (RT-PCR), and Enzyme-linked Immunosorbent Assay (ELISA) that is considered to be hallmarks of HERVs expression. It was observed that on the application of HERV-K peptides, more accumulation of interferon-gamma (IFN-γ) and tumor necrosis factor (TNF-α) was observed which clearly states that HERV-K has a stronger ability to modulate the immune system by stimulating various immune moderators that are involved in proinflammatory responses. HERV expression depends upon various factors, i.e., microbial infections and inflammations. In various pathological and physiological conditions, increased expression of HERVs was observed; therefore, various studies illustrated the involvement of one or more HERVs in certain diseases, i.e., neurological diseases.

Douville R et al. [153] identified the HERV in ALS patients’ neurons via RT-PCR. A study found increased expression of HERV-K pol transcripts in ALS patients in comparison to control. Sequencing revealed that the pol transcript frequency was high in the motor cortex while in the cortical neurons, reverse transcriptase protein was localized in a significant amount.

A transgenic mouse was created that can develop ALS-like pathophysiology, i.e., motor dysfunction, significant loss of lower and upper motor neurons, specific loss of motor cortex volume, and other injuries along with the potency to express HERV-K env gene. The study confirmed the contribution of HERV-K env protein in neurodegeneration [154].

Manghera et al. [155] performed an in vitro investigation of neuronal cells and astrocytes of ALS brain tissues and found that TDP-43 a protein member of DNA-RNA binding protein helps the protein deposition of HERV-K reverse transcriptase in neuronal cells. Both astrocytes and neuronal cells have different capabilities to clear and express protein deposition of HERV-K reverse transcriptase during proteasome inhibition and inflammation. In comparison to neuronal cells, astrocytes cleared the deposition more effectively via autophagy and stress granule formation.

HERV-K (subtype HML-2) reactivation strongly correlates with ALS pathophysiology, therefore, inhibiting HML-2 in ALS via antiretroviral therapy seems a possible solution to treat ALS patients. M. Garcia-Montojo et al. [156] explained that in vivo administration of antiretroviral therapy on HML-2 patients results in slower progression of clinical ALS symptoms.

In addition, ALS HERVs were also found to be associated with MS [157]. Multiple sclerosis-associated Retrovirus (MSRV) was identified as the first HERV released from cerebrospinal fluid (CSF) leptomeningeal cells in MS patients. MSRV has extracellular RNA along with a tryptophan-specific t-RNA binding site due to which it served as the base of the new HERV family named HERV-W. To date, several groups demonstrated the expression of HERC-W and the presence of MSRV particles in the brain, blood, and CSF of multiple sclerosis patients. Alongside increased expression of HERV-W was also detected in brain cells via flow cytometry on circulating monocytes, natural killer cells, macrophages, and B cells.

Epstein–Barr virus (EBV) is a lymphotropic herpes virus. Various studies confirmed the role of EBV in multiple sclerosis through mechanistic and epidemiological evidence. It is regarded as putative driver of MS. EBV infection increased the risk of MS up to 32 folds and more with HLA-DR2b and other several mononucleosis infections [158]. It is not elucidated how these environmental and genetic factors influence MS; however, clinical studies using cell-based, antiviral, and vaccine approaches targeting EBV confirmed its role as a putative driver of the disease [159].

Through the above-mentioned data, it is concluded that multifactorial experimental techniques have recognized the diverseness of bacterial and viral populations in CNS with various autoimmune neurological diseases. This diversity in each individual could serve as the basis of differences in clinical symptom evolution and severity.

### 10.2. Microbiota Dysbiosis in Autoimmune Neurological Diseases

Microbial dysbiosis has been regarded as a change in the profile of healthy human microbiota towards pathogenic and maladaptive commensal microbial communities [160,161]. The transfer of healthy microbiota into a more detrimental and pathogenic profile results in an altered immune reaction that ultimately leads to various diseases, i.e., multiple sclerosis (MS), amyotrophic lateral sclerosis (ALS), Parkinson’s disease (PD), Alzheimer’s disease (AD), etc., [162,163,164].

Current findings strongly highlight the importance of healthy gut microbiota in the function, development, and healthy aging of the brain [165]. Several helpful clues are collected from animal studies showing the role of healthy microbiota in preventing autoimmune neurological diseases. In ALS patients, gastrointestinal pathologies may be due to enhanced gut permeability compared to healthy controls, which results in an increased level of LPS with overexpressed monocytes production [166]. Another study found a decline in membrane permeability of the blood–brain barrier (BBB) and the blood–spinal cord barrier (BSCB) in ALS patients rather than in healthy controls [167,168].

Reduced membrane permeability of BBB results in enhanced immune cell infiltrations as well as intensified central nervous system inflammation [167]. Graves et al. [169] showed that impedes the integrity of BBB results in increased infiltration of immune cells, i.e., macrophages. Overexpression of COX-2 levels and mast cells was also observed.

Wu et al. [170] found the reduced abundance of *Butyrivibro fibrisolvens*, *Firmicutes* *peptostreptococcus*, and *E. coli* in the feces of ALS patients, which depicts the altered colonization of gut microbiota. Similarly, ALS mice exhibited upregulation of IL-17 along with systemic inflammation. Concludingly, the data suggested the role of microbial dysfunction in the pathology of ALS patients through the permeability of the CNS barrier, stimulation of systemic inflammation as well as deficiencies in the intestine.

In addition to the role of gut microbiota in ALS, studies also found the association with AD [171]. Enhanced intestinal permeability leads to activation of monocytes along with higher LPS levels in Alzheimer’s patients [166] while reduced BBB integrity results in activation of the chronic neuroinflammatory state alongside induction of microglia [172]. Gut microbiota dysbiosis along with BBB permeability results in enhanced adverse signaling in the cells of CNS and gut microbes. Moreover, studies also confirmed the systemic inflammation and endothelial and epithelial permeability results due to microbial dysbiosis in AD patients [173].

Significant evidence collected through these studies suggested that microbial dysbiosis can influence the pathophysiology of neurodegenerative diseases therefore these studies could lead to finding potential therapeutic methods that stabilize and enrich the gut microbiota. Through this, there will be a reduction or delay in autoimmune neurodegenerative disease prevalence.

#### 10.2.1. The Impact of the Gut–Brain Axis, Gut Microbiota, and Probiotics in AINDs

**Figure 4:**
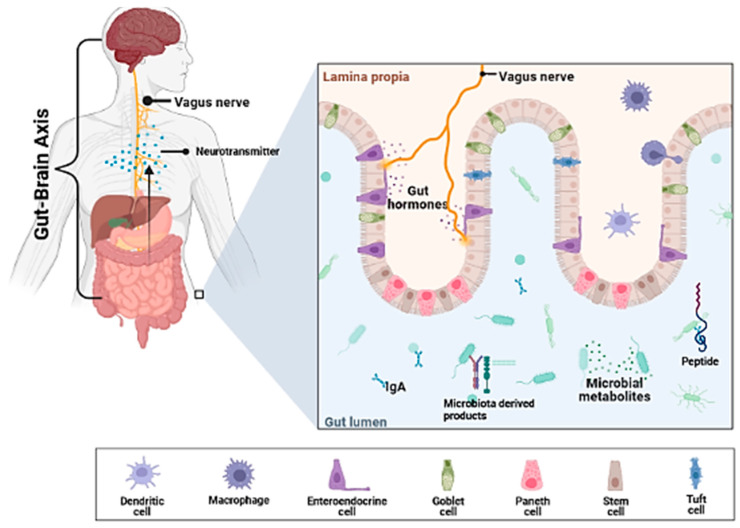
Gut–brain axis.

Numerous biochemical and interlinked hormonal pathways are associated with the health of the gastrointestinal tract (GIT) to the brain and are, therefore, regarded as an influential therapeutical possibility for probiotic usage against autoimmune neurodegenerative diseases. One potent and broad anti-inflammatory action can control the specifically linked microbiota with AINDs. Antigen-presenting cells including macrophages and dendritic cells are embedded in GIT subepithelial lamina propria tissue. Therefore, this placement brings immune cells into close vicinity to the gut microbiota, seized antigen and pathogens that cross the defensive epithelial barrier allow sufficiently communication between the immune system and environment [174]. Microbe-associated molecular patterns (MAMPs) of microbes can be recognized by NOD-like receptors (NLRs) and Toll-like receptors (TLRs) present on immune cells which in turn activate the signaling cascade a key to the expression of pro-inflammatory or anti-inflammatory cytokines (Figure 4) [175,176]. This is regarded consequential as neurodegeneration is associated with neuroinflammation. Moreover, the microbiota can communicate with host physiology, i.e., lipogenesis, apoptosis, insulin control, and hormonal and neuronal signaling through the production of secondary metabolites.

A variety of neuroprotective molecules are produced from commensal microbiota that can directly or indirectly influence the signaling pathways in the central nervous system [177]. Additionally, considerable molecular and endocrine signaling cascades interlink the brain and gut that regulate crucial processes. Some biomolecules obtained from the gut influence molecular and hormonal signaling pathways and are considered crucial for AINDs development.

#### 10.2.2. Ferulic Acid (FA)

Ferulic acid (FA) is a phenolic molecule ample in vegetables, i.e., carrots and tomatoes, fruits, i.e., oranges and pineapples, and in seed plants, i.e., oranges and pineapples. Conventionally, herbs and plants rich in FA are used as medicinal plants due to their anti-inflammatory and ROS scavenging abilities [178,179]. Present-day ferulic acid is regarded as a potent antioxidant molecule due to its potency to scavenge ROS and having the therapeutic ability to treat various illnesses such as cancer, diabetes, obesity, and neurodegeneration diseases. Various in vivo and in vitro studies confirmed the neurodegeneration abilities of FA via its ability to stimulate neural stem cell proliferation and direct influence on neuronal cells. In modern medicine, FA has been shown as a crucial target for moderating the linkage between brain and commensal microbiota. Aside from dietary uptake, FA is also synthesized by the FA esterase gene present in some microbiota species, i.e., *B. animalis* [180] and *L. fermentum* NCIMB 5221 [181]. In gut microbiota, certain species have feruloyl esterase enzymes that help in hydrolysis and release of ferulic acid from its bound state indicating the importance of microbiota in the action of FA.

Substantial research found the linkage between the therapeutic potential of FA and AD pathology. Through in vivo and in vitro AD models it is confirmed that FA has the potential to inhibit Aβ-related toxicity by impeding β-secretase activity and Aβ aggregation. Oral administration of ferulic acid in AD mice results in β-carboxy-terminal amyloid precursor protein (APP) cleavage, reduction in Aβ fibril formation, oxidative stress, and neuroinflammation [182]. In another AD mouse model, FA appeared to impair the amyloid deposition and Aβ1–42-induced memory and learning deficiency [183]. In a similar transgenic AD administration model, FA in combination with octyl gallate (OG) was found effective to improve cognitive behavior, reduction in β-amyloid plaques, enhancing α-secretase activity, increasing cleavage of amyloid β-protein precursor (APP), and inhibiting β-secretase activity. Moreover, synaptotoxicity, neuroinflammation, and oxidative stress also attenuated strikingly [184].

#### 10.2.3. Manipulation of AINDs through Microbiota Produced Short-Chain Fatty Acids

Several metabolites are formed by microbiota via fermentation of carbohydrates such as fructooligosaccharides and galactooligosaccharides. Fermentation is mostly mediated by *Bifidobacterium*, *Eubacterium*, *Roseburia*, *Bacteroides*, *Lactobacillus*, and *Propionibacterium,* which results in the production of metabolites including butyrate, acetate, and propionate short chain fatty acid (SCFAs) [185]. The kind of SCFA production depends upon the microbiota community in the gut and the type of consumed fiber [186]. SCFAs are known to influence the gut microbiota profile through endocrine signaling. To date, numerous studies confirmed the neuroprotective role of SCFAs, i.e., propionate and butyrate SCFAs have the potency to regulate catecholamines production by controlling gene expression of tyrosine hydroxylase in addition to dopamine biosynthesis [187]. This is considered crucial in PD pathology as tyrosine hydroxylase is often downregulated in the affected patient’s substantia nigra region which in turn effect dopamine synthesis. Another experimental study confirmed the neuroprotective role of propionic acid and butyric acid against AD patients due to their potential to downregulate amyloid beta A4 protein. Moreover, due to the histone deacetylase (HDAC) activity of butyrate and increased learning-associated gene expression, improvement in memory function was improved in a late-stage AD mouse model [188,189].

#### 10.2.4. The Impact of Gut Microbiota Producing Histamine in AINDs

Microbiota-producing histamine is identified as among the promising pharmacotherapeutic agents in controlling AINDs significantly in AD and MS [190]. It is directly synthesized from certain species of *Lactobacillus* and regarded as a biogenic monoamine compound that plays a substantial role in various physiological functions notably, wound healing, neurotransmitter, regulation of immune cells, allergic reactions, and cell proliferation [191]. Throughout the CNS there is a broad spectrum of histamine receptors particularly in the hippocampus, striatum, amygdala, thalamus, substantia nigra, and other parts that specify the extensive effect of histamine in the neuronal system. Histamine can act as a pro-inflammatory or an anti-inflammatory depending upon the receptor it acts upon [190,192]. It can create inflammatory responses in the brain by enhancing various chemokines and cytokines, i.e., IL-6, IL-1α and IL-1β production.

Recently, histamine was found as a gut metabolite produced from microbiota species, i.e., *Pediococcus*, *Lactococcus*, *Lactobacillus*, *Enterococcus*, and *Streptococcus as* they have histidine decarboxylase gene [193]. The inflammatory feature of histamine plays an important role in the pathogenesis of various AINDs particularly in MS and AD [193]. Regarding neurodegeneration, a higher level of histamine is correlated with AD as it increases nitric acid amount, which promotes neuroinflammation. A study found that in an experimental EAE mouse model, the permeability of BBB changes because of histamine leads to neuroinflammation because of the high influx of infiltered cells in CNS. Thus, histamines receptor can serve as an important drug target. However, studies found that exogenous histamines can stimulate remyelination by facilitating or inducing mature oligodendrocytes development, enhancing myelin formation, and by increasing progenitor cells migration into inflammatory sites [194].

## 11. Conclusions

Autoimmune neurological diseases constitute an area in the medical field regarded as the having adequate availability of pharmaceutical alternatives. The current pipeline of autoimmune neurological diseases comprises a surfeit of potential drugs with novel and diverse mechanisms of action although clinical potential has somehow been restricted. Various approved drugs are routinely used for treatment, but the unique therapeutic methodologies will demand the usage of rational drug combinations or multitarget drugs. However, the pathophysiology of autoimmune disorders is not completely understood and, hence, it might be anticipated that the pharmacotherapy of neurological diseases will remain that way without biomedical research in the future.

## Figures and Tables

**Table 1:** Advantages and Limitations associated with Immunomodulatory Drugs.

Advantages and Limitations of Immunomodulatory Drugs	
Advantages	Limitations
A combination of drugs within nutraceuticals has clinical advantages for the treatment of infections [44].	Immunomodulator drugs increase the risk of infection as they cause both mild and adverse effects on human health [45].
Nanomaterials in combination with molecular targeted therapy enhanced the immunomodulatory effect [46].	The major limitations related to immunomodulatory drugs are in vivo toxicity, routes of administration, and suitable formulations.
The combinations of vitamin D3 and phenylbutyrate activate innate immunity [47] and it produces antimicrobial peptides which can be used for the treatment of tuberculosis [48] as they produce both immunomodulatory and antibacterial responses.	Medullar suppression is caused by using immunomodulators such as azathioprine and 6-mercaptopurine. It is also recommended to take gastric protectors to avoid possible gastric irritation.
The major advantage of using immunomodulators is their well-known mechanism of action and long-term side effects [49].	Side effects of using immunomodulators are pancreatitis, dizziness, hepatitis, and myalgia [50].
A combination of drugs within nutraceuticals has clinical advantages for the treatment of infections [44].	Immunomodulator drugs increase the risk of infection as they cause both mild and adverse effects on human health [45].
	The major limitations related to immunomodulatory drugs are in vivo toxicity, routes of administration, and suitable formulations.

## Data Availability

Data sharing not applicable.
